# Semaphorin4A Is Cytotoxic to Oligodendrocytes and Is Elevated in Microglia and Multiple Sclerosis

**DOI:** 10.1177/1759091415587502

**Published:** 2015-05-25

**Authors:** Dominique F. Leitner, Bozho Todorich, Xuesheng Zhang, James R. Connor

**Affiliations:** 1 Department of Neurosurgery, Penn State University College of Medicine, Hershey, PA, USA; 2 Duke Eye Center, Department of Ophthalmology, Duke University School of Medicine, Durham, NC, USA; 3 Department of Pediatrics, Penn State University College of Medicine, Hershey, PA, USA

**Keywords:** H-ferritin, iron, multiple sclerosis, oligodendrocyte, Sema4A, Tim2

## Abstract

We have previously established that T cell immunoglobulin and mucin domain containing 2 (Tim2) is an H-ferritin receptor on oligodendrocytes (OLs). Tim2 also binds Semaphorin4A (Sema4A). Sema4A is expressed by lymphocytes, and its role in immune activation is known; however, its relationship to diseases that are known to have myelin damage has not been studied. In this study, we demonstrate that Sema4A is cytotoxic to OLs in culture: an effect accompanied by process collapse, membrane blebbing, and phosphatidylserine inversion. We further demonstrate that Sema4A preferentially binds to primary OLs but not astrocytes: an observation consistent with the lack of expression of Tim2 on astrocytes. We found that Sema4A protein levels are increased within multiple sclerosis plaques compared with normal-appearing white matter and that Sema4A induces lactate dehydrogenase release in a human OL cell line. The chief cellular source of Sema4A within the multiple sclerosis plaques appears to be infiltrating lymphocytes and microglia. Macrophages are known to express Sema4A, so we interrogated microglia as a potential source of Sema4A in the brain. We found that rat primary microglia express Sema4A which increased after lipopolysaccharide activation. Because activated microglia accumulate iron, we determined whether iron status influenced Sema4A and found that iron chelation decreased Sema4A and iron loading increased Sema4A in activated microglia. Overall, our data implicate Sema4A in the destruction of OLs and reveal that its expression is sensitive to iron levels.

## Introduction

We have recently established that ferritin is a major source of iron for oligodendrocytes (OLs) and can replace transferrin in culture media — the latter once considered as an obligate factor for OL survival in culture (Bottenstein, 1986). Ferritin uptake by OLs is mediated by the T cell immunoglobulin and mucin domain containing 2 (Tim2) receptor (Todorich et al., 2008). In addition to ferritin, Tim2 reportedly binds Semaphorin4A (Sema4A) which is expressed by T cells (Kumanogoh et al., 2002). Sema4A is a part of the Class IV semaphorin protein family, which are transmembrane proteins that can be cleaved into soluble peptides that function in the immune system, neuronal migration, and angiogenesis (Mizui et al., 2009; Yukawa et al., 2010; Meda et al., 2012). Thus, there is the potential for a neuroimmune interaction between Sema4A and the Tim2 receptor.

In the immune system, Sema4A is expressed by dendritic cells, B cells, activated T cells, and macrophages (Kumanogoh et al., 2002; Meda et al., 2012). It is involved in costimulating helper T cell proliferation and cytokine production (Kumanogoh et al., 2005). Mice deficient in Sema4A have dendritic cells with poor allostimulatory activities and impaired Th1 differentiation of T cells with consequent deficient Th1 responses (Kumanogoh et al., 2005). There have been suggestions in the literature that Sema4A could be part of a neuroimmune system axis. Blocking antibodies to Sema4A result in decreased clinical and histopathological severity in the mouse model of multiple sclerosis (MS), experimental autoimmune encephalomyelitis, which is a prototypical Th1-mediated autoimmune disorder (Kumanogoh et al., 2002). Additionally, elevated levels of Sema4A in serum have been associated with Th17 skewing in MS patients (Nakatsuji et al., 2012).

Because the direct effect of Sema4A has not been characterized at the level of the OL, we evaluated whether Sema4A binds to these cells and influences their cell viability. Sema4A has been implicated in MS, so we also evaluated its expression in MS plaques. Moreover, because peripheral macrophages have increased expression of Sema4A upon activation (Meda et al., 2012), we determined whether Sema4A is expressed by microglia from the brain and whether its expression changes after activation. Because iron homeostasis in microglia is altered after activation, we evaluated whether iron dyshomeostasis influenced Sema4A expression in microglia and whether OL viability decreases in the presence of Sema4A.

## Methods

### Recombinant Proteins

Recombinant Sema4A-Fc, Sema4D-Fc, and Sema3A-Fc proteins were obtained from R&D Systems (Minneapolis, MN). The extracellular domains of these proteins were produced and purified as Fc-chimeras in a mammalian cell line. Apo-transferrin was purchased from Sigma (St. Louis, MO). Recombinant H-ferritin (Hft) was prepared in competent BL21 *Escherichia coli* as described previously (Todorich et al., 2008). Human anti-IgG1 HRP secondary antibody (Abcam, Cambridge, MA) was used against the IgG-fused domain to Sema4A-Fc, Sema4D-Fc, and Sema3A-Fc at 1:5,000.

### Primary Cultures

Sprague Dawley rat pups were used for primary cell cultures. Mixed glia were subcultured and then subjected to sequential purification using the shaking method to obtain cultures enriched for each glial subtype (McCarthy and de Vellis, 1980). Rats were maintained in accordance with the National Institute of Health Guide for the Care and Use of Laboratory Animals and according to the Pennsylvania State University Institutional Animal Care and Use Committee guidelines. Briefly, newborn rat pups from postnatal Day 1 to 2 were decapitated and sterilized in 70% ethanol. Brains were dissected and washed in Hank’s Balanced Salt Solution (HBSS). After removing blood vessels and meninges, brain tissue was incubated with trypsin-ethylenediaminetetraacetic acid for 20 min at 37℃. Tissue was then passed through a 135 -µm nylon mesh, followed by two 40 -µm meshes. Cells were plated in 10% fetal bovine serum (FBS)/Dulbecco’s Modified Eagle’s medium (DMEM) with 1 × antimycotic–antibiotic and glucose. Thirteen days after plating, microglia were shaken off at 265 rpm for 1.5 hr at 37℃. Oligodendrocyte progenitor cells (OPCs) were removed by shaking an additional 18 hr with further purification by allowing contaminating cells to adhere to a cell culture Petri dish. OPCs were then plated into poly-D-lysine-coated plates in N2S media. N2S contains DMEM/F12, 0.5% FBS, 0.66 g/L bovine serum albumin, 10 mg/L D-biotin, 40 mg/L apo-transferrin, 16 mg/L putrescine, 6 µg/L progesterone, 1 × penicillin-streptomycin-glutamine, 1 × insulin-transferrin-selenium-ethanolamine solution (ITS-X), 10 µg/L platelet-derived growth factor, and 25 µg/L fibroblast growth factor. OPCs were differentiated in N2B2 media, which contains N2S media with platelet-derived growth factor and fibroblast growth factor removal. This protocol promotes maturation of OLs including expression of myelin basic protein (MBP; data not shown). Cells were allowed to differentiate for 4 days. OL-enriched fractions were plated and examined by bright field microscopy, which showed a typical yield of over 90% enriched OLs. The cultures were then trypsinized to remove the astrocytes and plated in six-well plates with DMEM/10% FBS media. Both sets of cells were used for experiments within 2 to 3 days after plating.

For the microglia experiments, cells were cultured for 2 days before experiments were performed. After microglia were plated, cells were examined by bright field microscopy and showed a typical yield of over 90% enriched microglia. Subsequently, they were treated with 0.8 µg/mL lipopolysaccharide (LPS, Sigma) and increasing concentrations of ferrous ammonium sulfate (FAS) or deferoxamine (DFO, Sigma) for 24 hr. Lysates were produced by washing cells with HBSS and incubating cells with Radio-Immunoprecipitation Assay buffer (RIPA buffer, Sigma) containing protease-inhibitor cocktail (Sigma). The solution was sonicated and centrifuged, and the supernatant was used. Bicinchoninic assay (Pierce, Rockford, IL) was used to determine protein content.

### Human OL Cell Culture

The human OL cell line MO3.13 (McLaurin et al., 1995; Buntinx et al., 2003) was used to evaluate the effect of Sema4A on cell cytotoxicity. The MO3.13 cell line was obtained from Cellutions Biosystems Inc. (Ontario, Canada), which was originally derived from the fusion of human temporal lobe OLs and human rhabdomyosarcoma cells. Immature cells were cultured in DMEM with 10% FBS and 1 × penicillin-streptomycin-glutamine. Mature cells were produced by differentiating immature cultures in DMEM containing 100 nM 4-b-phorbol 12-myristate 12-acetate (Sigma), 1 × penicillin-streptomycin-glutamine, and no FBS for 3 days.

### Cytotoxicity Assays

Increasing concentrations of recombinant Sema4A-Fc were added directly to the media of either rat OLs or astrocytes. For the MTT assay (3-(4,5-dimethylthiazol-2-yl)-2,5-diphenyltetrazolium bromide assay), cells were exposed to recombinant Sema4A for 20 hr, and the MTT reagent was added for the last 4 hr of treatment. Cells were solubilized in sodium dodecyl sulfate (SDS)-based buffer overnight at 37℃, and subsequently, absorbance was measured at 595 nm using Spectramax Gemini plate reader.

Annexin V binding was evaluated as a measure of cell cytotoxicity. OPCs were incubated with recombinant Sema4A-Fc at the K_D_ concentration of 10 µg/mL for the cleaved recombinant dimer, which has also been used in other studies (Yukawa et al., 2010). Cells were washed in ice-cold phosphate-buffered saline (PBS, pH = 7.4) and then exposed to annexin V-conjugated to AlexaFluor488 dye (Invitrogen, Carlsbad, CA) at 1:5 dilution in annexin V binding buffer (pH = 7.4) for 15 min (Invitrogen). The cells were subsequently washed 2 times in annexin V binding buffer and then visualized on a Nikon inverted fluorescent microscope equipped with a digital camera. Images in random fields were acquired, and cells were counted in four quadrants by an experimenter blinded to the experimental condition.

To further evaluate cytotoxicity, lactate dehydrogenase (LDH) release was quantified from human OL cells (MO3.13) to determine the response to Sema4A. Increasing concentrations of recombinant Sema4A-Fc were added to the cell culture media for 24 hr. For the LDH assay (Roche), LDH catalyst was added to the cell culture media and incubated for 15 min at room temperature. Absorbance was read at 492 nm using the Spectramax Gemini plate reader (Molecular Devices, Sunnyvale, CA).

### Binding Studies

#### Saturation binding study

To evaluate binding of Sema4A, recombinant Sema4A-Fc was labeled with iodine-125 (I^125^), using the method of Hunter and Greenwood (1962) as previously described (Todorich et al., 2008). Primary rat OPCs and astrocytes were plated in six-well plates at a concentration of 1 million cells per well. Media was removed, and cells were washed with HBSS supplemented with MgCl_2_ and CaCl_2_. Increasing concentrations of rSema4A-Fc-I^125^ protein were dissolved in PBS buffer (pH = 7.4) and were incubated with primary rat OPCs or astrocytes for 2 hr at 22℃. After three washes in PBS buffer, cells were harvested mechanically in RIPA buffer, and counts were measured with a gamma counter. Subsequently, the protein concentration of the counted sample was determined with a BioRad DC assay (Hercules, CA), and all counts were normalized to total protein in each of the samples.

#### Competition binding study

Media was removed from primary rat OPCs in six-well plates at a density of 1 million cells per well and subsequently washed with HBSS supplemented with MgCl_2_ and CaCl_2_. The cells were then preincubated with increasing concentrations of rSema4A-Fc (recombinant Semaphorin4A-Fc) protein, recombinant Hft, or apo-transferrin for 2 hr at 4℃ in PBS buffer (pH = 7.4). Next, rSema4A-Fc-I^125^ was added to reach a final concentration of 0.1 µg/mL and incubated for 1.5 hr at 22℃. The binding suspension was removed, and cells were washed 3 times with PBS buffer. Cells were harvested mechanically, and counts were measured on the gamma counter.

### Immunostaining of the MS Plaques

Paraffin-embedded blocks of MS plaque tissue were obtained from the Human Brain and Spinal Fluid Resource Center (VA West Los Angeles Healthcare Center, Los Angeles, CA; sponsored by NINDS/NIMH, National Multiple Sclerosis Society, Department of Veterans Affairs). Paraffin-embedded normal tissue controls that were age- and sex-matched were obtained from Harvard Brain and Tissue Resource Center (Belmont, MA; sponsored by grant from National Institutes of Health, R-24-MH 068855). All sections were cut at 5 µm thickness and subsequently deparaffinized in xylenes and hydrated in ethanol. After a brief rinse in dH_2_O, antigen retrieval was performed in 10 mM citrate buffer (pH = 6.0) for 20 min. Subsequently, endogenous peroxidase activity was blocked in 3% H_2_O_2_/methanol and then blocked in 5% milk/PBS (pH = 7.4). The sections were incubated with either anti-Sema4A 1:100 (rabbit polyclonal; Abcam) for 16 hr at 4℃ or anti-MBP 1:200 (mouse monoclonal; Upstate) for 16 hr at 4℃. After several washes in PBS, sections were incubated in secondary antibodies (ABC Vectastain kit) in accordance with the manufacturer’s protocol. The staining was visualized with diaminobenzidine using standard procedure. For MBP staining, diaminobenzidine was intensified with nickel chloride solution. All images were acquired on a bright field microscope equipped with a digital camera.

Paraffin-embedded blocks of MS tissue were also used for colocalization of Sema4A with immune cell markers. As mentioned earlier, all sections were cut at 5 µm thickness and subsequently deparaffinized in xylene and dehydrated in ethanol. Sections were blocked in 5% milk in PBS for 1 hr, followed by primary antibody incubation overnight at 4℃. Anti-Sema4A 1:100 and anti-CD11b 1:50 (rat, BD Pharmingen) or CD3 1:50 (mouse; Abcam) were used in 5% milk. Subsequently, sections were washed in PBS and were incubated with secondary antibody for 2 hr at room temperature in 5% milk with FITC 488 rabbit (Invitrogen) 1:200 and RITC 555 mouse (Invitrogen) 1:200. The anti-CD11b primary antibody was conjugated to Cy7. 4′,6-diamidino-2-phenylindole at 1:1,000 was included with secondary antibody incubation. Sections were washed in PBS and coverslipped. Images were acquired on a fluorescent microscope equipped with a digital camera.

### Western Blots

Denaturing SDS-PAGE (BioRad) gels were used to separate 8 µg of microglia protein per lane. Proteins were transferred to nitrocellulose, which was subsequently blocked with 5% milk Tris-buffered saline tween-20. Blots were probed for Sema4A, Hft, or beta-actin for 16 hr at 4℃. Anti-Sema4A (1:200, rabbit polyclonal antibody) was purchased from Abcam. Anti-Hft (1:1,000, rabbit monoclonal antibody) was purchased from Cell Signaling (Danvers, MA). Anti-beta-actin (1:3,000, mouse monoclonal antibody) was purchased from Sigma. Corresponding anti-rabbit or anti-mouse HRP-conjugated secondary antibodies were used (1:5,000, GE Amersham, Amersham, UK). Protein was visualized with ECL reagents (Perkin–Elmer, Waltham, MA) on the FujiFilm LAS-3000 System and Image Reader LAS-3000 software. Densitometry quantification was performed using MultiGauge software.

### Statistical Analysis

Graph Pad 4 was used to perform statistical analyses as indicated.

## Results

**Figure 1.**
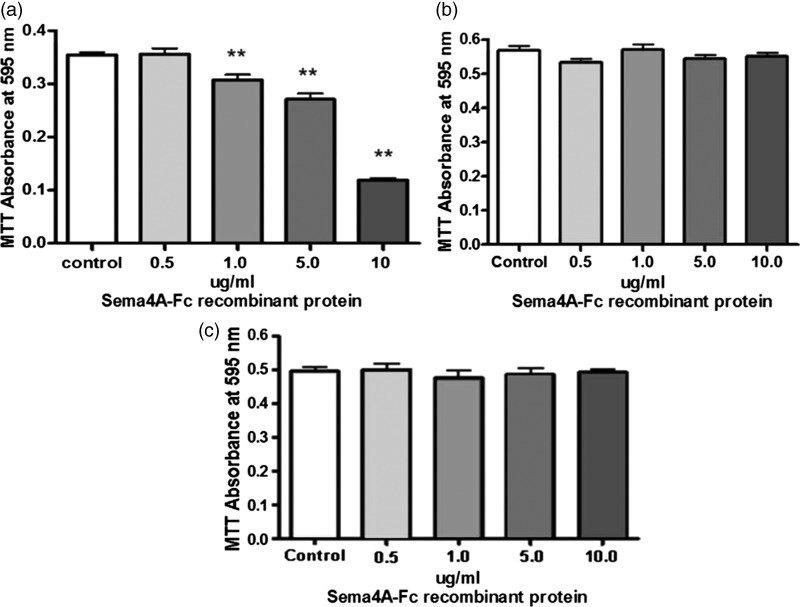
Sema4A is cytotoxic to primary OPCs but not astrocytes in culture. Purified cultures of primary rat OPCs, astrocytes, and mature oligodendrocytes were treated with increasing concentrations of recombinant Sema4A-Fc protein for 24 hr. Untreated cells served as a control. Cell viability was determined with an MTT assay. Individual means were evaluated for statistical significance using one-way analysis of variance with Dunnett’s posttest comparisons with the control group. The results, which are representative of three independent experiments, demonstrate dose-dependent cytotoxicity of primary OPCs (a) but not astrocytes (b) or mature oligodendrocytes (c) to Sema4A protein. OPC = oligodendrocyte progenitor cell. **p < .01.

To investigate the biological effects of Sema4A on OLs, we used the cell culture model of primary rat OPCs. First, we incubated purified cultures of primary rat OPCs and astrocytes with increasing concentrations of recombinant Sema4A-Fc for 24 hr in order to determine whether Sema4A had any cytotoxic effects. Recombinant Sema4A-Fc exposure resulted in dose-dependent cytotoxicity to OPCs but not to astrocytes or mature OLs (Figure 1). Specifically, the 24-hr incubation with 10 µg/mL rSema4A-Fc resulted in cytotoxicity and a loss of over 50% of the OPCs under the experimental conditions used in our study.

**Figure 2.**
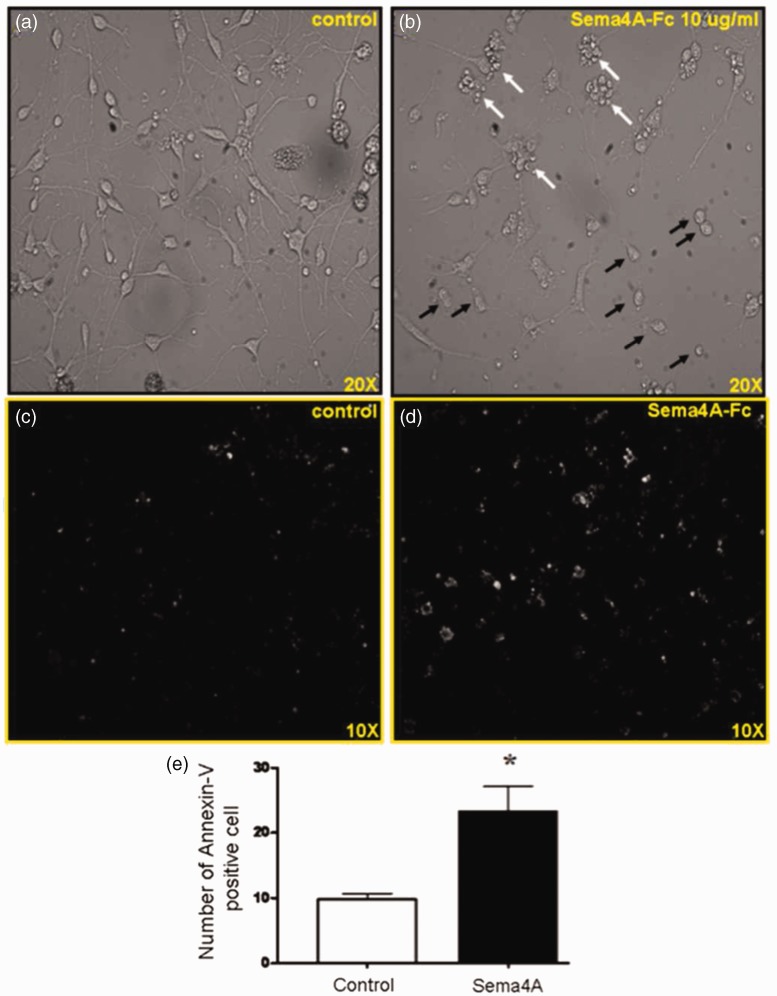
Sema4A collapses processes and causes apoptosis of oligodendrocyte progenitors. (a, b) Primary rat OPCs were incubated with 10 µg/mL Sema4A-Fc protein for 8 hr. The untreated cells served as a control. Controls show the typical morphology of OPCs, which were spindle-shaped bipolar cells with extended and ramified processes (a). On the other hand, Sema4A-treated OPCs show process collapse (black arrows), while others appear as grape-like clusters suggestive of apoptotic membrane blebbing (white arrows) (b). Apoptosis was confirmed by Annexin V labeling in a separate experiment. Control cells (c) exhibit weak labeling with annexin V, while Sema4A treatment results in a significant increase in the number of annexin V-reactivity in the Sema4A-treated group (d). Cell counts reveal a 2.5-fold increase in annexin V labeling as a result of Sema4A treatment. Means were analyzed for significance using the unpaired *t* test (e).

Morphologically, compared with the untreated controls in Figure 2(a), collapse of processes is noted in the rSema4A-Fc-treated OPCs as early as 8-hr posttreatment (Figure 2(b)). Furthermore, many of the cells in the rSema4A-Fc-treated groups had grape-like membrane blebbing suggestive of cytotoxicity. To further confirm rSema4A-Fc-induced cytotoxicity, we performed annexin V labeling of rSema4A-Fc-treated and control OPCs (Figure 2(c) and (d)). Annexin V dye binds with high avidity to phosphatidylserine, an inversion of which to the outer leaflet of plasma membrane is an early event at the initiation of apoptosis. After 8 hr, rSema4A-Fc-treated OPCs demonstrated a 2.5-fold increase in Annexin V labeling compared with control, suggesting that rSema4A-Fc-induced cytotoxicity is indeed via activation of apoptotic pathways (Figure 2(e)).

**Figure 3.**
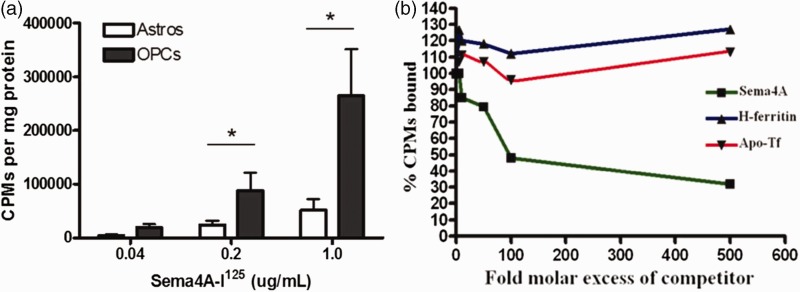
Sema4A binds to oligodendrocyte progenitors but not astrocytes in culture. (a) Purified cultures of primary rat OPCs and astrocytes were incubated with increasing concentrations of Sema4A-Fc-I^125^, and after thorough washes, the cells were harvested and radioactive counts measured, normalized to total protein per sample. The means were evaluated for significance with a one-sided *t* test. Data are representative of two independent experiments. (b) Preincubating primary OPCs with increasing concentrations of cold Sema4A-Fc but not rH-ferritin or transferrin effectively competes Sema4A-Fc-I^125^ binding. Data are representative of two independent experiments. CPM = counts per minute.

To define whether specific binding sites existed for Sema4A on OLs, we radiolabeled rSema4A-Fc protein with iodine-125 (I^125^). In Figure 3(a), we demonstrate that rSema4A-Fc binding to purified cultures of OPCs had approximately a fivefold higher avidity than to primary rat astrocytes, suggesting that receptors for rSema4A-Fc are expressed by OPCs and not astrocytes. To demonstrate that this binding is specific, we performed a competition binding experiment. Figure 3(b) demonstrates that increasing concentrations of cold unlabeled rSema4A-Fc protein but not recombinant Hft or apo-transferrin, were able to inhibit the binding of rSema4A-Fc-I^125^ to OPCs. These data suggest that Sema4A binds specifically to receptors on OLs but that the binding domains for rSema4A-Fc on OPCs are distinct from those of Hft and transferrin.

**Figure 4.**
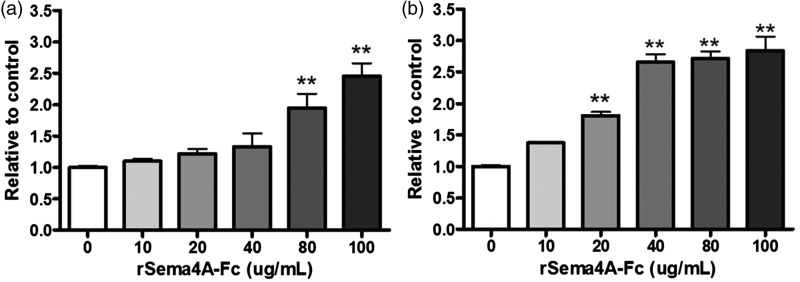
Sema4A is cytotoxic to immature and mature human oligodendrocytes *in vitro*. Immature and mature MO3.13 human oligodendrocytes were treated with increasing concentrations of recombinant Sema4A-Fc protein for 24 hr. Untreated cells served as a control. Cell toxicity was determined by LDH release. Individual means were evaluated for statistical significance using one-way analysis of variance with Dunnett’s post hoc comparisons. The results demonstrate dose-dependent cytotoxicity to Sema4A protein in immature oligodendrocytes (a) and mature oligodendrocytes (b). Values represent average ± *SEM*. **p < .01.

Next, we evaluated the human OL cell line MO3.13 for sensitivity to Sema4A. Exposing the cells to increasing concentrations of rSema4A-Fc resulted in increasing cytotoxicity to both immature and mature cells (Figure 4(a) and (b)), although the mature cells were affected at a lower concentration of Sema4A than the immature cells.

**Figure 5.**
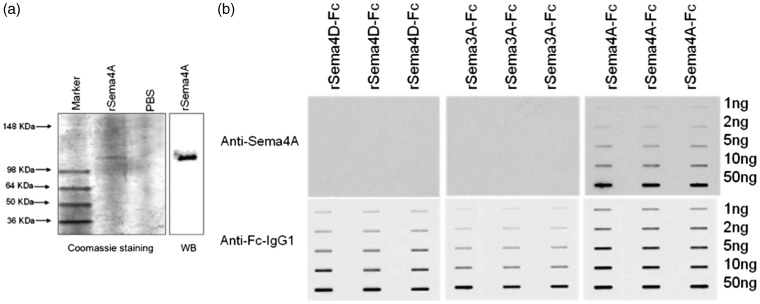
Anti-Sema4A antibody recognizes Sema4A but not closely related Sema3A or Sema4D. (a) Recombinant Sema4A-Fc chimera protein was loaded onto a denaturing SDS-PAGE gel and either stained with Coomassie or probed with anti-Sema4A antibody on a Western blot. The antibody recognizes the Sema4A-Fc chimera recombinant protein, which is of appropriate molecular size (115–120 kDa) for the glycosylated protein. PBS (0.1 M) was loaded as a negative control. (b) Native (non-denatured) rSema4A-Fc, rSema3A-Fc, and rSema4D-Fc were immobilized onto a nitrocellulose membrane and probed with anti-Sema4A antibody via slot blot. A separate blot was probed with anti-IgG antibody against the fused Fc domain of the semaphorin proteins to show total protein. This representative immunoblot demonstrates that the Sema4A antibody recognizes rSema4A-Fc but not rSema3A-Fc or rSema4D-Fc. WB = Western blot.

**Figure 6.**
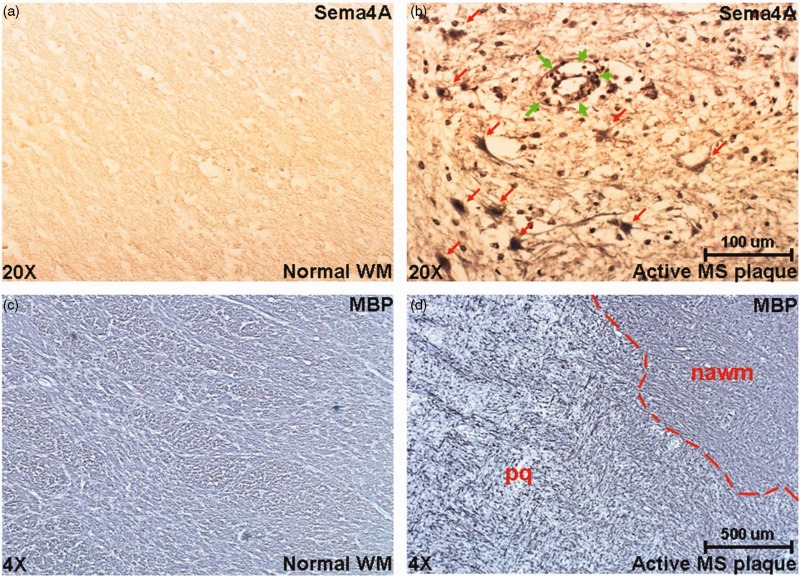
Sema4A is expressed in MS plaques. Sections of normal white matter (a) and those of patients with MS (active plaques, b) were immunostained for Sema4A. Representative images are presented that demonstrate strong immunoreactivity with Sema4A antibodies in the MS plaque compared with normal white matter. The reaction product was mainly localized in lymphocytes (perivenular cuff, green arrows in b) and in scattered macrophages/microglia (red arrows in b). The size and location of the plaque was determined by immunostaining of the neighboring sections for myelin basic protein (c and d). These low-powered images demonstrate representative results of myelin basic protein immunoreactivity in both normal white matter and MS plaques. Within the given specimen, the demarcation between the region of strong demyelination/plaque (pq) and normal-appearing white matter (nawm) was observed and is indicated by the red dotted line. MS = multiple sclerosis; WM = white matter.

**Figure 7.**
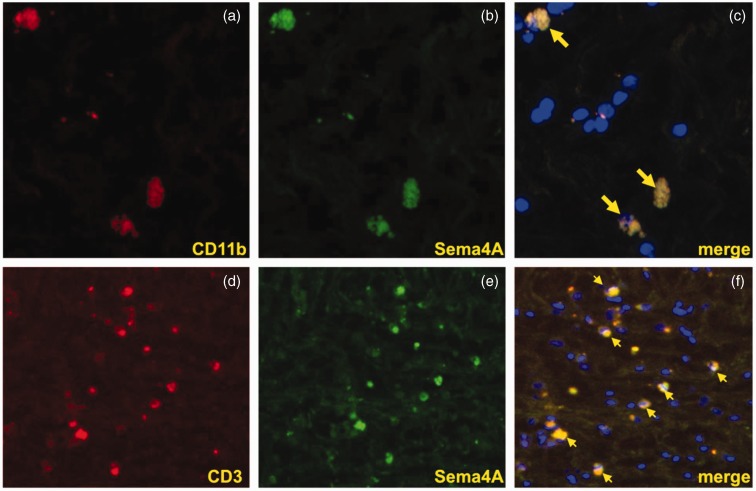
Sema4A colocalizes with CD11b and CD3 in MS plaques. (a and b) Sema4A (green) is detected within cells that are positive for the microglia/macrophage marker CD11b (red) in MS plaques. (c) In the merged image, arrows indicate cellular colocalization of both CD11b and Sema4A. (d–f) The T cell marker CD3 (red) is detected within MS plaques. Sema4A immunostaining (green) reveals cells of similar appearance to the T cells and the merged image (f) identifies (arrows) cellular colocalization of CD3 and Sema4A. Representative images are depicted at 40 × magnification.

To investigate the expression of Sema4A protein in MS plaques, we first demonstrated that the Sema4A antibody recognizes Sema4A recombinant protein on a Western blot (Figure 5(a)). Because Sema3A and Sema4D have been suggested to play a role in the pathogenesis of MS and are expressed within MS plaques (Williams et al., 2007), we demonstrated that this antibody did not cross-react with these related semaphorin proteins on a native protein immunoblot (Figure 5(b)). This immunoblot shows that anti-Sema4A antibody recognizes only native Sema4A-Fc but not Sema3A-Fc or Sema4D-Fc. Equal loading of protein is shown with an antibody against the IgG-fused domain to rSema4A-Fc, rSema4D-Fc, and rSema3A-Fc. Subsequently, we used the same antibody to evaluate expression and cellular distribution of Sema4A in five MS patient plaques and four control specimens containing normal human white matter. Table 1 summarizes the histopathological parameters of the specimens used in this study. The MS and control patient population were matched well by age (average age 67 and 68, respectively) and gender (male/female ratio was 20%:80% for MS and 25%:75% for controls). In Figure 6, a representative image of immunostaining for Sema4A demonstrates much higher levels of Sema4A immunoreactivity within the plaque compared with normal white matter. Sema4A immunoreactivity was localized in small cells cuffing the blood vessels, which is pathognomonic for infiltrating lymphocytes within the MS plaque. A second group of Sema4A-positive cells observed in the sections were large bloated cells infiltrated within the myelin. These cells are morphologically typical of microglia/macrophages. The identity of these cells was confirmed by colocalization of Sema4A with a lymphocyte marker (CD11b) or macrophage marker (CD3; Figure 7). In each of five plaques examined, the Sema4A immunoreactivity was much higher in the plaque compared with normal white matter. We defined the extent of demyelination and distinguished plaque area from normal-appearing white matter by staining for MBP (Figure 6(c) and (d)) for each plaque used in the study. Collectively, these data demonstrate that there are higher levels of Sema4A protein within the MS plaque compared with normal white matter, and that the chief cellular sources of this protein within the plaque are infiltrating lymphocytes and activated macrophages/microglia.

**Table 1.** Characteristics of Multiple Sclerosis Patients and Normal Controls Used in the Study.

	Primary diagnosis	Age	Gender	Plaque	Pathological classification
MS patients					
1	MS	76	F	Active	Demyelination, lymphocytic infiltrate (E), microglia present
2	MS	84	F	Active	Demyelination, lymphocytic infiltrate (D), microglia present
3	MS	65	M	Active	Demyelination, lymphocytic infiltrate (P, E), microglia present
4	MS	62	F	Active	Demyelination, lymphocytic infiltrate (E, D), microglia present
5		48	F	Active	Demyelination, lymphocytic infiltrate (P, E, D), microglia present
Control patients					
1	Normal	66	M	None	Normal white matter
2	Normal	49	F	None	Normal white matter
3	Normal	77	F	None	Normal white matter
4	Normal	80	F	None	Normal white matter

*Note.* The tissue specimens from a total of nine patients were used in the study, of which five were MS patients and four were normal controls. The average age of MS patients and controls were 67 and 68 years, respectively. MS = multiple sclerosis.

**Figure 8.**
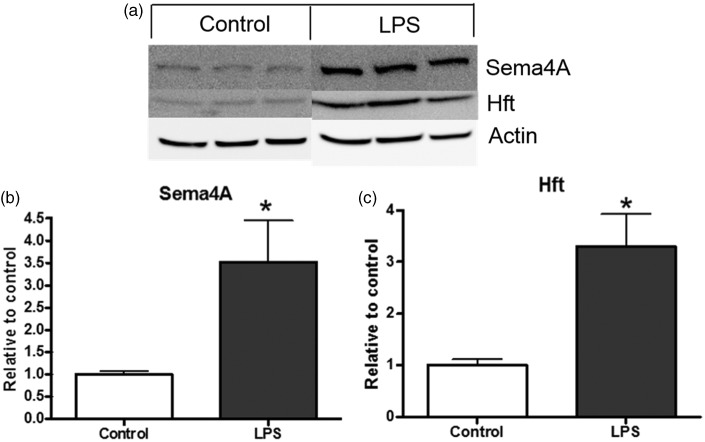
Sema4A is increased after microglial activation with LPS. (a and b) Sema4A is expressed by rat primary microglia and is increased upon activation with 0.8 µg/mL LPS for 24 hr. Hft is also increased after LPS treatment, indicating activation of the microglia. Data are representative of three independent experiments, completed in triplicate. Protein quantification is relative to beta-actin loading control. Values represent average ± *SEM*. Student’s *t* test was used to determine significance. LPS = lipopolysaccharide; Hft = H-ferritin. *p < .05.

We next interrogated microglia as a potential source of Sema4A protein in the brain because macrophages in the periphery express Sema4A (Meda et al., 2012) and because microglia and macrophages are associated with MS plaques. Using rat primary cultures of microglia, we found that microglia express Sema4A under resting conditions in culture (Figure 8(a)). To determine whether activation influenced this expression, we incubated the microglia with LPS for 24 hr. When compared with the untreated control cells, Sema4A was significantly increased by LPS activation by almost fourfold (*p* < .05, Figure 8(a) and (b)). We also evaluated the expression level of Hft after LPS treatment as a positive control for activation (Weiss, 2002; Schonberg and McTigue, 2009). Hft was significantly increased after LPS exposure by about 4.5-fold (*p* < .05, Figure 8(a) and (b)). This finding shows that not only do microglia express Sema4A, but they also upregulate the expression of Sema4A after activation.

**Figure 9.**
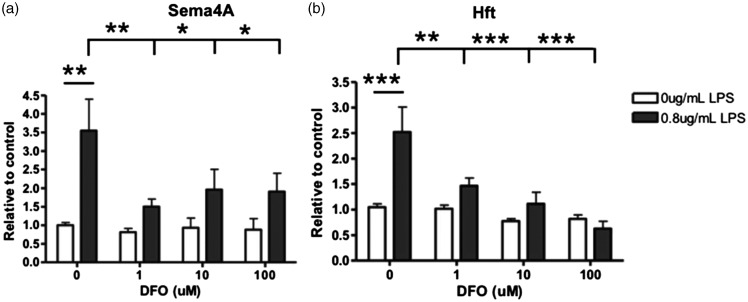
Microglial expression of Sema4A protein was decreased after DFO induced iron deficiency while activated by LPS. Primary rat microglia cells were treated with increasing DFO or with 0.8 µg/mL LPS and increasing concentrations of DFO for 24 hr. (a) Sema4A is significantly increased after LPS alone. With the addition of LPS/DFO, there is a significant decrease in Sema4A expression. (b) Hft expression is significantly increased after LPS alone. With the addition of LPS/DFO, Hft expression is significantly decreased. Protein quantification is relative to beta-actin loading control. Two-way analysis of variance with Bonferroni post hoc test was used to determine significance. Values represent average ± *SEM*. DFO = deferoxamine; LPS = lipopolysaccharide; Hft = H-ferritin. *p < .05; **p < .01; ***p < .001.

Iron status influences many cellular functions, including microglial activity (Cheepsunthorn et al., 2001b; Weiss, 2002; Beard et al., 2003; Todorich et al., 2009; Mairuae et al., 2011; Rathnasamy et al., 2011). Therefore, we investigated whether iron chelation or iron loading influenced the expression level of Sema4A protein by microglia. First, we found that increasing concentrations of iron chelator DFO resulted in no significant effect on Sema4A and significantly decreased Hft expression (Figure 9). With LPS alone, Sema4A and Hft protein expression was increased (Figure 9), but DFO decreased the effect of LPS on Sema4A expression (Figure 9(a)). A similar observation was made for the combined effect of LPS and DFO on Hft expression (Figure 9(b)). This indicates that restricted iron availability to microglia reduces the production of Sema4A.

**Figure 10.**
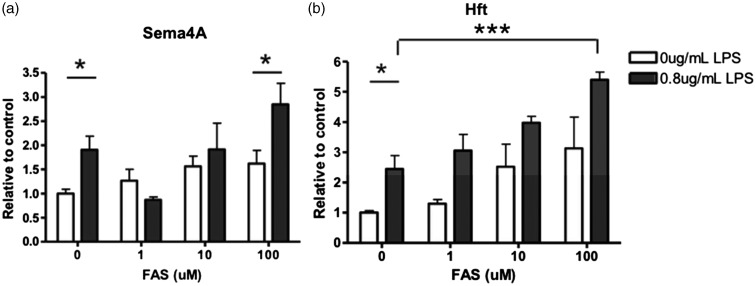
Microglial expression of Sema4A protein was increased after FAS induced iron loading while activated by LPS. Primary rat microglia cells were treated with increasing FAS or with 0.8 µg/mL LPS and increasing concentrations of FAS for 24 hr. (a) Sema4A is significantly increased after LPS alone. With the addition of LPS/FAS, there is a significant increase in Sema4A expression. (b) Hft expression is significantly increased after LPS alone. With the addition of LPS/FAS, expression is significantly increased. Protein quantification is relative to beta-actin loading control. Two-way analysis of variance with Bonferroni post hoc test was used to determine significance. Values represent average ± *SEM*. FAS = ferrous ammonium sulfate; Hft = H-ferritin. *p < .05; **p < .01; ***p < .001.

Cellular iron loading induced by FAS (Figure 10(a) and (b)) did not influence Sema4A but did increase Hft expression. With increasing concentrations of FAS in the presence of LPS, there was a significant increase in Sema4A expression (Figure 10(a)). Hft expression was also significantly increased with increasing concentrations of FAS in the presence of LPS (Figure 10(b)). These data indicate a synergistic effect of iron and LPS activation on Sema4A expression in microglia.

## Discussion

In this study, we demonstrate that the Class IV neuroimmune semaphorin, Sema4A binds to and is cytotoxic to OLs as shown by phosphatidylserine inversion and LDH release. Sema4A-induced death of OPCs is accompanied by process collapse, membrane blebbing, and phosphatidylserine inversion, which is highly suggestive of apoptosis. We also demonstrated that Sema4A is strongly expressed in MS plaques compared with normal white matter, and that within the plaques, Sema4A expression primarily is localized to infiltrating lymphocytes and microglia/macrophages. In a cell culture model, we demonstrated that microglia express Sema4A and that the Sema4A expression in these cells can be enhanced by an inflammatory agent (LPS), and the levels of expression are iron responsive. The latter is an important finding because microglia accumulate iron when activated (Cheepsunthorn et al., 2001a; Urrutia et al., 2013). Our findings suggest that Sema4A is a significant contributor to OL death in neuroimmune demyelinating disease. The findings reported in this study reveal a novel pathway in the neuroimmune axis and have implications for therapeutic options in the treatment of demyelinating disorders.

Our findings for Sema4A are similar to a report by Williams et al. (2007), who reported that two Class III semaphorins, Sema3A and Sema3F, are also richly expressed within actively demyelinating MS plaques. Collectively, our results contribute to the evolving concept that various semaphorins are active participants of the inflammatory signal plex within MS plaques and that local immune cells may participate in the pathological signaling to destroy OLs involving these molecules.

Although originally described as axonal chemorepellants in the developing central nervous system, semaphorins have been shown recently to regulate a variety of physiological and pathological functions within and outside of the central nervous system. We have shown in this study that at least one of the biological functions of Sema4A in OPCs is regulating their survival through the induction of apoptosis. The role of various semaphorins in regulating apoptosis has been well documented. For example, Sema3A promotes apoptosis of leukemic T cells (Moretti et al., 2008), neurons through Plexin A3 (Ben-Zvi et al., 2008), and activated microglia (Majed et al., 2006). Sema3F acts synergistically with Sema3A in promoting apoptosis of endothelial cells (Guttmann-Raviv et al., 2007). Sema3B induces apoptosis of breast and lung cancer cells (Castro-Rivera et al., 2004; Castro-Rivera et al., 2008).

Finally, Sema4D (CD100) has been shown to collapse processes and cause apoptosis of neural precursors and immature OLs (Giraudon et al., 2004). Giraudon et al. have also demonstrated that Sema4D is overexpressed within the inflammatory demyelinating lesions of HAM/TSP (HTLV-1 associated myelopathy/tropical spastic paraparesis) patients and that Sema4D-overexpressing T cells result in process collapse and destruction of immature OLs but not astrocytes. Therefore, our results taken in concert with those of Giraudon et al. suggest that both Class IV semaphorins, Sema4A and Sema4D, have very similar biological functions on OLs. Although both Sema4A and Sema4D are transmembrane proteins, direct cellular contact with the target cell is apparently not essential for their biological activity, as extracellular domains of both of these proteins fused to Fc are sufficient to exert the biological effects of the parent protein (Kumanogoh et al., 2002; Giraudon et al., 2004; Toyofuku et al., 2007). This is further reinforced by the finding that soluble Class IV semaphorin proteins are released by proteolytic cleavage of the extracellular domain that is dependent on cellular activation (Wang et al., 2001). Which of the semaphorin forms (soluble vs. membrane bound) predominates *in vivo* in MS plaques is to be determined. We did not find secretion of Sema4A in the microglial cultures even after iron loading and activation with LPS (data not shown). The absence of detectable Sema4A in the media suggests cell to cell contact between microglia, and OLs may be necessary for Sema4A to exert its effect similar to that reported for T cells (Kumanogoh et al., 2002). A secreted form of Sema4A is likely to come from lymphocytes that are infiltrating the plaque area, and a soluble form of Sema4A has been reported in serum of MS patients (Nakatsuji et al., 2012).

As mentioned, the proposed mechanism by which Sema4A may be inducing apoptosis is through receptors on OLs. Sema4A reportedly binds to the Tim2 receptor in the immune system (Kumanogoh et al., 2002; Wilker et al., 2007), and antibodies to Sema4A reduce the severity of neurological symptoms in the experimental autoimmune encephalomyelitis demyelinating animal model. We have previously demonstrated that Tim2 is selectively expressed by OLs in rodents and that it acts as a cellular receptor for Hft, an iron delivery protein (Todorich et al., 2008). In that study, we showed that Tim2 is expressed by CNPase, O4, and A2B5-positive OLs but not glial fibrillary acidic protein -positive astrocytes. Similarly, in this study, Sema4A binding follows a similar trend whereby it is much higher in OLs compared with astrocytes. However, we could not compete off Sema4A binding on OPCs with recombinant Hft. Therefore, it is possible that Sema4A and Hft bind to different domains of Tim2, and they might not compete for the same site of the receptor. Additionally, it has been demonstrated that Sema4A can bind to B-type plexin receptors and Plexin D1 on endothelial cells (Toyofuku et al., 2007). Plexin B3 is detected in postnatal OLs (Worzfeld et al., 2004; Worzfeld et al., 2009) and binds Sema5A (Artigiani et al., 2004) and Sema4A on COS7 cells (Yukawa et al., 2010). These studies raise the possibility that a member of either the B- or D-type plexins may also be a cellular receptor of Sema4A on OLs.

In conclusion, our findings clearly demonstrate that Sema4A is toxic to OLs. Moreover, there is a relationship between iron status and Sema4A expression in microglia. In a neuroimmune disease with demyelination and neuroinflammation, Sema4A is elevated in the brain. The involvement of Sema4A in MS pathogenesis provides a therapeutic target for the treatment of not only MS but also, potentially in other cases, neuroinflammation leading to demyelination.

## Summary

Sema4A is cytotoxic to oligodendrocytes and is increased in MS plaques. Microglia express Sema4A upon activation and could be the source of Sema4A in the brain. The expression of Sema4A by microglia is iron responsive.
